# A formal analysis of *Listeria monocytogenes* cross-contamination dynamics in multi-species biofilms

**DOI:** 10.1038/s41538-025-00535-3

**Published:** 2025-08-08

**Authors:** Raquel A. Nogueira, Juan J. Rodríguez-Herrera, José Luis López-Carmona, Antonio Valero, Marta L. Cabo

**Affiliations:** 1 Laboratory of Microbiology and Technology of Marine Products (MICROTEC), Instituto de Investigaciones Marinas (IIM), Consejo Superior de Investigaciones Científicas (CSIC), Vigo, Spain; 2 Departamento de Bromatología y Tecnología de los Alimentos, Grupo de Investigación en Higiene Bromatológica (HIBRO), UIC Zoonosis y Enfermedades Emergentes (ENZOEM), International Agrifood Campus of Excellence (CeiA3), Universidad de Córdoba, Córdoba, Spain

**Keywords:** Biofilms, Microbial communities, Pathogens

## Abstract

Food contamination by *Listeria monocytogenes* usually occurs in food processing environments, where bacteria coexist in multi-species biofilms. Using a stochastic modelling approach, this study addressed the cross-contamination dynamics of cold-smoked salmon by *L. monocytogenes* from multi-species biofilms (3) formed by bacterial consortia from food industry surfaces under conditions reproducing low *L. monocytogenes* loads. Single-species biofilms were also formed for comparison. Transfer rates (TR) were determined over successive contacts of each biofilm with 25 salmon slices. Modelling of TR probabilities revealed distinct contamination profiles. Differences were particularly marked between multi- and single-species biofilms, likely indicating the influence of the coexisting microbiota. Subsequently, the growth of *L. monocytogenes* in cold-smoked salmon after transfer from multi-species and single-species biofilms was examined. *L. monocytogenes* from multi-species biofilms showed higher growth than single-species biofilms, which would markedly increase food safety risk. These findings are critical for designing realistic challenge studies and improving contamination control of ready-to-eat foods.

## Introduction

*Listeria monocytogenes* is the causative agent of listeriosis, a foodborne illness with a fatality rate of up to 20–30%^1–3^, making it the subject of health concerns and surveillance and causing significant economic losses. Listeriosis can be particularly life-threatening in the population groups known as Young, Old, Pregnant, and Immunocompromised people^4^, and it is mainly caused by consuming contaminated ready-to-eat (RTE) food^5,6^. These products pose a significant food safety risk due to the absence of a final bactericidal treatment before consumption^7^, with high occurrences in meat and fish commodities, such as smoked salmon^8^. Despite intensified surveillance and control measures, the notification rate has increased consistently in the last few years^3^, indicating a recurrent presence of the pathogen in foods.

The presence of *L. monocytogenes* in foods is related to its ability to tolerate adverse environmental conditions, such as low pH^9^, low temperatures^10^, high salt concentrations^11^, desiccation^12^ and the presence of antimicrobials^13^, among others. Food contamination frequently occurs in food processing environments (FPE), where L*. monocytogenes* usually coexist with other bacteria in multi-species biofilms^14^. The biofilm provides a favourable environment to survive under adverse conditions, while facilitating nutrient acquisition and exchange of genetic material^15^. Thus, biofilms serve as reservoirs for *L. monocytogenes*, increasing the likelihood of cross-contamination of foods^16–18^. Also, biofilms may colonise areas on equipment or installations that are difficult to access due to poor design^19^, which often impedes the effective action of disinfectants. Accordingly, cross-contamination research has progressively moved from planktonic cells^20–22^ to biofilm systems^23–25^. However, studies have primarily focused on single-species and, to a much lesser extent, dual-species systems, which represent simplified models of the complex multi-species biofilms commonly present in real FPEs, leaving the influence of coexisting microbiota largely unexplored.

Therefore, this study aimed to examine the cross-contamination of cold-smoked salmon with *L. monocytogenes* from multi-species biofilms found in the food industry. For this purpose, typical conditions of FPEs were simulated, using low initial loads of *L. monocytogenes* and low nutrient availability. The study included: (i) a comparative analysis of transfer dynamics from three different multi-species biofilms systems; (ii) a comparison of transfer dynamics of *L. monocytogenes* from three different multi-species biofilms; (iii) a comparison of transfer dynamics between multi-species and single-species biofilms of *L. monocytogenes* to a the influence of coexisting microbiota on contamination; and (iv) an assessment of the growth potential of *L. monocytogenes* transferred from both types of biofilm during the shelf life of smoked salmon, complemented by a case study illustrating how this growth potential could impact the risk of listeriosis associated with the consumption of smoked salmon.

## Results

### Dynamics of cross-contamination from multi-species biofilms

**Fig. 1:**
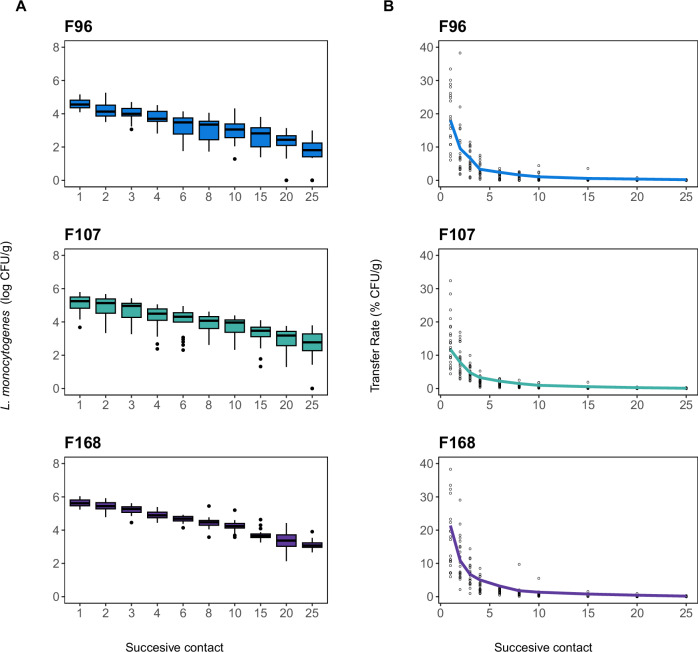
Transfer dynamics of *L. monocytogenes* to smoked salmon by 25 successive contacts with multi-species biofilms (F96, F107 and F168). **A** Box plots for the number of cells transferred at each contact. **B** Transfer Rate (TR) at each contact. Experimental values are shown as open circles, whereas solid lines correspond to estimates obtained with a Weibull model.

The numbers of *L. monocytogenes* cells transferred to smoked salmon from the multi-species biofilms under study, namely F96, F107 and F168, decreased progressively with each successive contact (Fig. 1A). Cell densities of *L. monocytogenes* varied significantly between biofilms, ranging from 10^4^ CFU/cm^2^ in F96 and F107 to 10^5^ CFU/cm^2^ in F168 (*p* < 0.05, Supplementary Table 1). Given those differences in the number of *L. monocytogenes* among the biofilms, transfer rates (TRs) were used to compare cross-contamination from the three biofilms.

A first approach based on pairwise comparisons revealed no significant differences in TR between biofilms in any of the 25 contacts (*p* > 0.05). Also, the dynamics of TR were analysed using three different models, namely, exponential decay^20,26^, logistic^25^ and Weibull^27,28^. Estimations and statistics from subsequent goodness-of-fit analyses of such models to the experimental values of TR showed that the Weibull distribution was the best fit for all three biofilms (Supplementary Table 2).

Based on the experimental data, a Monte Carlo method was used to generate data simulations, and their means were fitted to a Weibull distribution, followed by a noise correction. Fitting and noise correction were iterated 10^4^ times to improve the accuracy of the model (Fig. 1B; for more information, see 'Methods'). A comparison of the model parameter values (shape and scale) obtained for each of the biofilms revealed no significant differences either (*p* > 0.05, Supplementary Table 3).

**Fig. 2:**
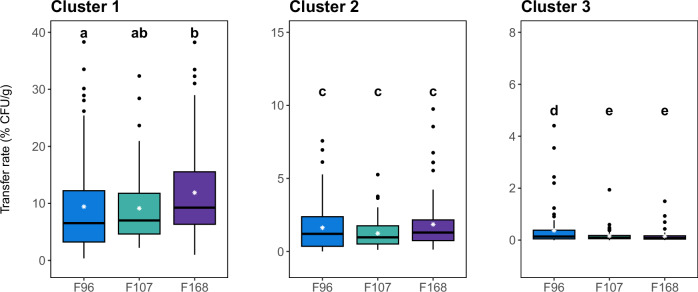
Box plots for transfer rates of multi-species biofilms (F96, F107 and F168) after contact clustering. The 25 contacts were clustered (1–3) based on the absence of significant differences in experimental values (*p* > 0.05); the contacts included in each cluster are listed in Table 2. Different letters in each cluster indicate significant differences between biofilms (*p* < 0.05).

Given the results, a stochastic approach was attempted, grouping contacts into different clusters to increase the number of replicates. The contacts of each biofilm were grouped into three clusters based on the absence of significant differences in experimental TR values (*p* > 0.05). As a result, the contacts within each cluster were the same for all three biofilms, except the third and tenth contacts, respectively, included in clusters 2 and 3 of F96 (Fig. 2 and Table 1). These three clusters could be associated with different stages of the transfer dynamics, namely, cluster 1, initial detachment; cluster 2, unstructured biofilm detachment; and cluster 3, the transfer of cells tightly attached to the inner layers of biofilms.

**Table 1:** Grouping contacts per biofilm system according to the Kruskal–Wallis test

	Contacts per biofilms system			
Cluster	L96	F96	F107	F168
Cluster 1	1, 2, 3 and 4	1, 2 and 3	1, 2 and 3	1, 2 and 3
Cluster 2	4, 6, 8 and 10	3, 4, 6, 8 and 10	4, 6, 8 and 10	4, 6, 8 and 10
Cluster 3	10, 15, 20 and 25	10, 15, 20 and 25	15, 20 and 25	15, 20 and 25

**Fig. 3:**
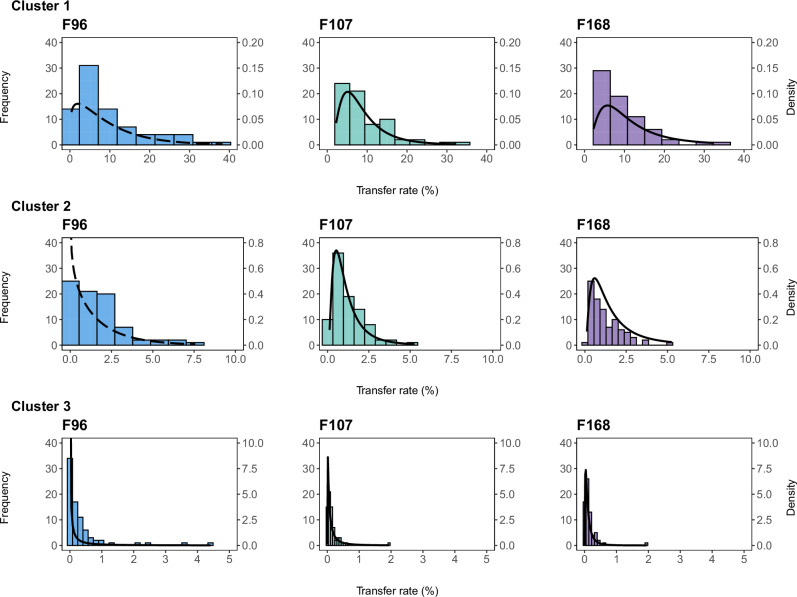
Frequency distributions of experimental TRs of the different contactclusters of each multi-species biofilm. The lines represent the probability density functions (PDF) of the best-fit distributions obtained for each case: gamma distribution (dashed line) for clusters 1 and 2 of F96 and log-normal distribution (solid line) for all others.

The experimental TR values of each cluster were then represented in histograms for each biofilm (Fig. 3), and differences in frequency distributions between biofilms were evaluated within each cluster by a Kolmogorov–Smirnov (KS) test. The tests revealed significant differences between F96 and F168 in cluster 1 (*p* = 0.01, *D* = 0.27), as well as between F96 and F107 (*p* = 0.02, *D* = 0.23) and F96 and F168 (*p* = 0.01, *D* = 0.27) in cluster 3. In contrast, no differences were found between biofilms in cluster 2. Subsequently, the experimental values of each of the nine clusters were fitted to log-normal, gamma, and Weibull distributions. The goodness-of-fit analysis showed that the gamma distribution provided the best fit for clusters 1 and 2 of F96, whereas the log-normal distribution was the best fit for the others (Supplementary Table 4).

The probability density functions (PDF) with best goodness-of-fit were then determined using the adjusted parameter values for each case. PDFs allow quantifying the probabilities (P) of transfer events directly related to the risk of cross-contamination. Accordingly, within cluster 1, a TR > 20% would be more likely to occur for F168 (*P* = 0.12) than for F96 (*P* = 0.08) or F107 (*P* = 0.09), indicating a higher risk of initial cross-contamination from that biofilm. In contrast, in cluster 2, the probability of a TR > 5% would be 0 for F168 but not for F96 (*P* = 0.09) or F107 (*P* = 0.01), whereas F96 would show the highest probability of TR > 1% in cluster 3 (0.12, versus 0.03 for F107 and 0 for F168). Therefore, the distribution analysis revealed differences between biofilms. While F168 showed the highest cross-contamination potential in the first stages of the dynamic (first cluster), F96 maintained high cross-contamination likelihoods throughout the 25 contacts.

### Transfer dynamics of single-species biofilms versus multi-species biofilms

A second set of transfer dynamics studies was conducted with single-species biofilms formed by the *L. monocytogenes* strains under study, namely L96, L107 and L168. A single assay was carried out to explore the ability of the three *L. monocytogenes* strains to form single-species biofilms under several low-nutrient conditions, specifically 1000-, 900-, 750- and 500-fold diluted TSB, and to find out which conditions would replicate the low loads of *L. monocytogenes* generally found in FPEs. The ability to form single-species biofilms under starvation varied significantly among the three strains, but a sharp decline in the number of biofilm cells of all strains was only found in 1000-fold diluted TSB, presenting a reduction of 2–5 orders of magnitude relative to 500-fold diluted TSB (Supplementary Fig. 1). Therefore, to replicate the low loads of *L. monocytogenes* generally found in FPEs^29^ and attempt to make them as similar as possible to those of multi-species biofilms, 1000-fold diluted TSB was used in single-species biofilm formation assays conducted subsequently. Subsequent trials with L107 and L168 did not detect attached cells; that is, the number of *L. monocytogenes* cells attached to the coupons was found to be below the detection limit of the method, i.e. less than 100 CFU per coupon (4 CFU/cm^2^). Therefore, the comparative analysis of transfer dynamics between single-species and multi-species biofilms was conducted only with strain L1.96.

As depicted in Fig. 5A, the transfer dynamics of *L. monocytogenes* were similar in both biofilm systems, displaying a decreasing pattern that reached very low values in the last contacts. Nonetheless, more cells were transferred from L96 than from F96, which was consistent with the initial load of *L. monocytogenes* in each biofilm (5.68 ± 0.27 log CFU/cm^2^ in L96 and 4.52 ± 0.68 log CFU/cm^2^ in F96). However, in terms of TR, significant differences were only observed between L96 and F96 first contact (*p* < 0.05), with higher values for F96, while all subsequent contacts showed similar values.

**Fig. 4:**
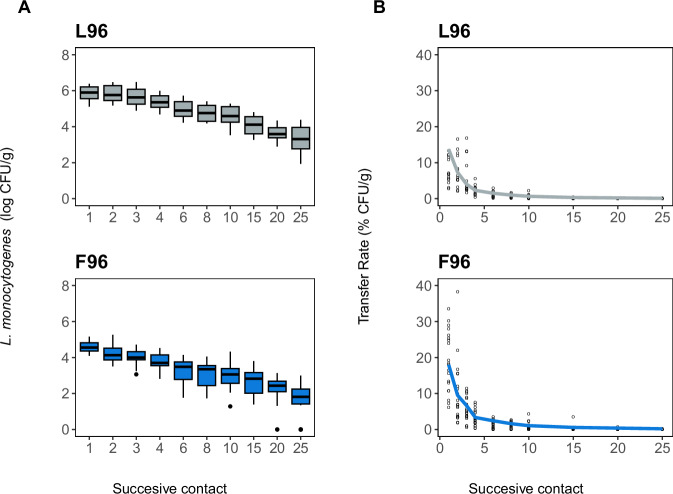
Transfer dynamics of *L.monocytogenes* to smoked salmon by 25 successive contacts with single-species (L96) and multi-species (F96) biofilms. **A** Box plots for the number of cells transferred at each contact. **B** Transfer rate (TR) at each contact. Experimental values are shown as open circles, whereas solid lines correspond to estimates obtained by fitting the Weibull model. A set of 20 replicates was used for each biofilm.

**Fig. 5:**
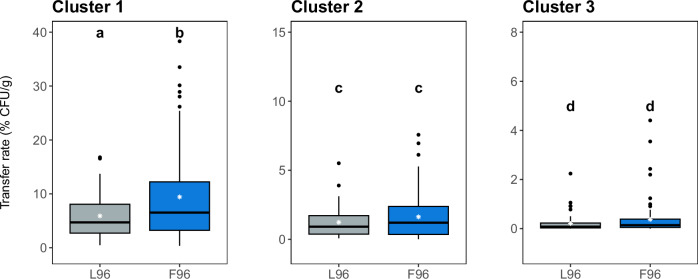
Box plots for transfer rates of the single-species (L96) and multi-species (F96) biofilms after contact clustering. The 25 contacts were clustered (1–3) based on the absence of significant differences in experimental values (*p* > 0.05); the contacts included in each cluster are listed in Table 2. Different letters in each cluster indicate significant differences between biofilms (*p* < 0.05).

Among the three distributions tested, it was observed that the Weibull distribution also provided the best fit for L96 (Fig. 4B and Supplementary Table 2). Similarly, the values obtained for the parameters of the final model approach did not reveal significant differences with those of F96 (Supplementary Table 3). The values of TR obtained for L96 were also clustered into three groups of contacts based on the absence of significant differences (*p* > 0.05, Fig. 5). The Kruskal–Wallis test reported differences only in cluster 1, in which L96 showed significantly lower TR than F96. Similar results were obtained when comparing frequency distributions in each cluster, with F96 and L96 only showing significant differences in cluster 1 (KS, *p* < 0.05, *D* = 0.2).

**Fig. 6:**
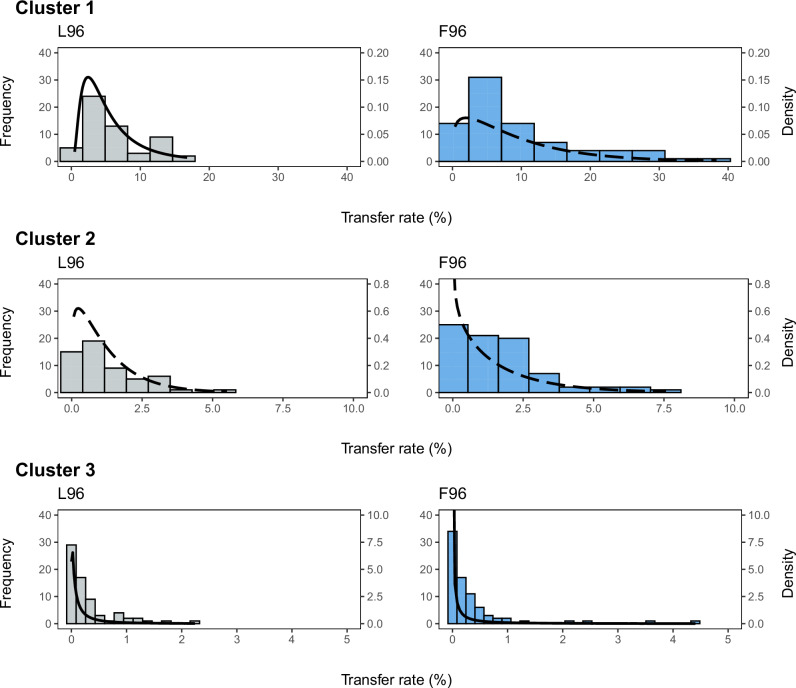
Frequency distributions of experimental TRs of the different contact clusters of the single-species(L96) and multi-species (F96) biofilms. The lines represent the probability density functions (PDF) of the best-fit distributions obtained for each case: gamma distribution (dashed line) and log-normal distribution (solid line).

Concerning the modelled PDFs, slight variations were found between L96 and F96 (Fig. 6). PDFs obtained for L96 consistently exhibited a narrower distribution with a lower degree of tailing in all clusters. This was particularly evident in cluster 1, with no TR higher than 20%. In terms of probabilities, differences were reported only for cluster 1, with P (TR > 20%) being 0 for L96 and 0.1 for F96. Thus, the cross-contamination potential of multi-species biofilm F96 was initially higher than the single-species counterpart L96.

### Risk of growth of *L. monocytogenes* from single-species and multi-species biofilms in cold-smoked salmon

**Fig. 7:**
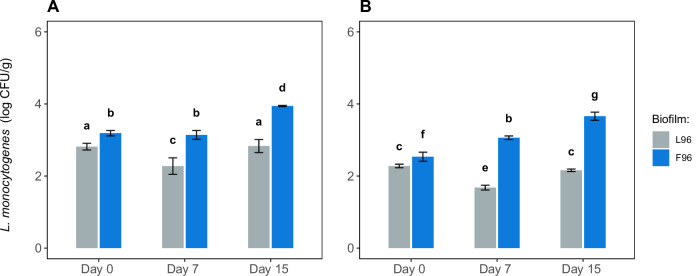
*L. monocytogenes* counts in cold-smoked salmon during storage at 5 °C cells were transferred from single- (L96) and multi-species (F96) biofilms. **A** Counts in portions contaminated at the first contact (cluster 1). **B** Counts in portions contaminated at the sixth contact (clusters 2 and 3). Different letters indicate statistically significant differences (*p* < 0.05).

Figure 7 shows the evolution of *L. monocytogenes* loads in smoked salmon contaminated with either F96 or L96 biofilms at the first and sixth contact over a 15-day storage period. Significant differences (*p* < 0.05) were observed between the two biofilm systems throughout the storage period. By the end of the storage period, *L. monocytogenes* growth from multi-species biofilms significantly exceeded that from single-species biofilms. For the first contact, loads reached 679.27 ± 252.33 CFU/g and 8764 ± 289.93 CFU/g for L96 and F96, respectively. Similar trends were observed for the sixth contact, with final loads of 144.19 ± 11.32 CFU/g and 4556.54 ± 1197.81 CFU/g for L96 and F96, respectively, indicating that smoked salmon contaminated with F96 presents a significantly higher load of *L. monocytogenes* than those slices contaminated with L96.

### Case study

**Fig. 8:**
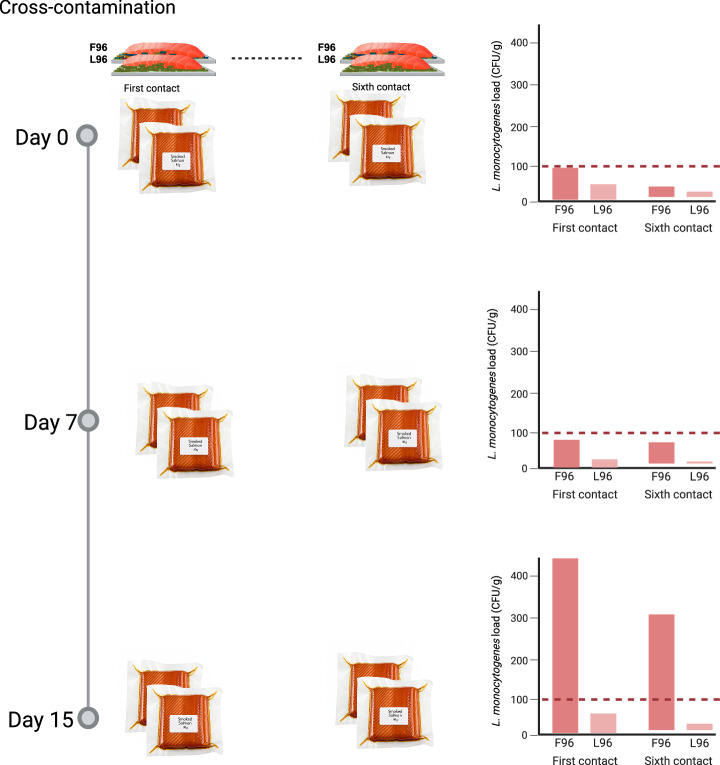
*L. monocytogenes* cross-contamination casa study in cold-smoked salmon. Estimates of *L. monocytogenes* load in cold-smoked salmon during storage at 5 ºC. In this specific case study, contamination is modelled to occur within a 5 g portion of a single slice in each 80 g package. *L. monocytogenes* was transferred at 1st or 6th contact from both single- (L96) and multi-species (F96) biofilms. Estimates were obtained from the experimental results of this study. The red dashed line marks the 100 CFU/g limit set in legislation. This image was created with BioRender.com.

Based on the results presented, a hypothetical scenario was simulated (Fig. 8). The simulation specifically involved a single smoked salmon slice (~5 g) within an 80 g package. Utilising the contamination data reported on single- and multi-species biofilms (Fig. 7), the microbial load transferred to a 5 g slice was calculated, and the final contamination level (CFU/g) was reported for the entire 80 g package.

According to the scenario results, the legal limit of 100 CFU/g *of L. monocytogenes* load would not be exceeded after 7 days of storage in both biofilm systems. However, by day 15, the limit would be significantly surpassed on multi-species biofilm, reaching 547.76 CFU/g and 284.78 CFU/g for the first and sixth contacts, respectively. In contrast, *L. monocytogenes* from single-species L96 remained below the detection limit in both contacts throughout the shelf life. Therefore, in this scenario*, L. monocytogenes* from multi-species biofilms poses a significantly higher risk than those from single-species biofilms.

## Discussion

Multi-species biofilms are highly complex and variable systems that are challenging to study. Numerous factors modulate the variability of the system, affecting biofilm structure and cell transfer dynamics. Many of these factors are difficult or impossible to control and replicate consistently in laboratory settings. Despite these challenges, investigating multi-species biofilms provides a more realistic representation of how bacteria, such as *L. monocytogenes*, interact and behave within the context of FPE, offering valuable insights into the dynamics of contamination events.

The similarity in the TR profiles of the three multi-species biofilms under study could be explained by the high variability, i.e. stochasticity, which makes it difficult to appreciate possible differences, unless they were large. In fact, differences among biofilms arose when the contacts were grouped into three clusters (thus increasing the number of TR data), as shown by the PDFs. To the best of the author’s knowledge, cross-contamination with *L. monocytogenes* from multi-species biofilms had not been addressed previously, which prevents any comparison with other works. The vast majority of studies on the issue have focused on single-species biofilms^24,25^ or at most, dual-species biofilms^23^. In dual-species biofilms, Pang and Yuk^23^ reported a TR of 10% at a single contact of smoked salmon with dual-species biofilms of *L. monocytogenes* and *Pseudomonas fluorescens*.

These studies represent simplified models of those complex multi-species biofilms commonly present in FPEs, leaving the influence of coexisting microbiota largely unexplored. Specifically, Pang & Yuk^23^ combined initial concentrations of 10^6^ CFU/mL of each *L. monocytogenes* and *P. fluorescens* to form dual-species biofilms. As a result, *L. monocytogenes* densities of 10^7^ UFC/cm^2^ were found in 48 h old biofilms. These loads are substantially higher than those generally found in FPE. Additionally, they relied on a small number of replicates (*n* = 6), which reduces the statistical power and the ability to detect meaningful effects. In contrast, we present a stochastic modelling approach based on using a high number of replicates (*n* > 60, after grouping contacts). By modelling contamination as a probabilistic process, this approach enables us to capture the inherent variability of contamination, enhance our ability to detect differences, and provide a more robust understanding of transfer dynamics.

The differences in the contamination profiles observed among the biofilms are probably due to the different microbiota present in each of them, rather than to the *L. monocytogenes* strains. Thus, the co-existing microbiota would probably determine the biofilm structure and cross-contamination potential. In this sense, Kim and Oh^30^ suggested that differences in cross-contamination of chicken meat and skin from dual-species formed either by *Salmonella enterica* and *Campylobacter jejuni* or *S. enterica* and *Clostridium perfringens* and multi-species biofilms formed by all three bacteria could be due to the different bacterial composition of each biofilm, which affects the attachment and production of extracellular polymeric substances (EPS). Similarly, a recent study has shown that the microscopic 3D structure of mixed-species biofilms is determined by the coexisting microbiota rather than the specific strain of *L. monocytogenes*^31^. In addition, it is well established that *L. monocytogenes* survives but does not outcompete other bacteria within biofilms^32^. Previous studies on multi-species biofilms harbouring *L. monocytogenes* showed that coexisting bacterial species outnumber the pathogen^29,33,34^. Similarly, surface sampling in FPEs revealed a low abundance of *L. monocytogenes* at contamination hotspots, in which many other bacterial species predominated in the microbial communities^35^. Accordingly, in our experimental systems, the inoculum of *L. monocytogenes* (10^2^ CFU/mL) was set three orders of magnitude below those of each coexisting bacterium (10^5^ CFU/mL) to reflect conditions found in industrial settings. However, little is known about the interactions of these bacteria with *L. monocytogenes* in biofilms, apart from enhancing its resistance against disinfectants^31,36^ and increasing *L. monocytogenes* counts in dual-species biofilms with *Acinetobacter* or *Pseudomonas*^37^. Therefore, further studies are needed to elucidate these complex interactions fully.

To gain further insight into the role of the coexisting microbiota in cross-contamination, single-species biofilms of *L. monocytogenes* were formed under the same conditions. Based on a screening experiment, a 1000-fold dilution of TSB in PBS was selected as the low-nutrient medium that better replicated the low microbial load of *L. monocytogenes* found in FPEs. Unexpectedly, strains L1.107 and L1.168 did not form single-species biofilms in 1000-fold diluted TSB, unlike previous screening results. The lack of experimental reproducibility observed for those two strains could be due to severe starvation and low inoculum. Conditions close to the growth/no-growth limit and low inoculum (10^2^ CFU/mL) have been reported to lead to experimental variations that are very difficult or even impossible to control^38^. Stochastic variations in growth kinetics are known to become apparent at low inoculum due to individual cell heterogeneity, which affects small populations much more than large ones^39^. Similarly, growth limits for planktonic *L. monocytogenes* cells were found to be distributed over a range of values and were affected by inoculum size^40,41^.

These findings contrast with the results obtained for multi-species biofilms, in which attached *L. monocytogenes* cells were found for the three strains. In fact, *L. monocytogenes* did not seem to experience growth limitations when coexisting with other bacteria. It has been noted that the presence of diverse metabolic pathways within multi-species biofilms could lead to metabolic cross-feeding^42^, as certain bacterial metabolites can serve as an energy source for other biofilm members. Recently, Ch’ng et al.^43^ have shown that *Enterococcus faecalis* benefits from the heme group produced by *Staphylococcus aureus* to use in aerobic respiration within dual-species biofilms. However, to the author’s knowledge, the specific metabolic interactions between *L. monocytogenes* and coexisting bacteria have not been addressed. Therefore, that possibility should be substantiated through future analyses evaluating microbial interactions within biofilms.

Since no cell counts were detected in single-species biofilms of L107 and L168, the comparison between single-species and multi-species biofilms was limited to those formed by the strain L1.96. Both the initial *L. monocytogenes* load in biofilms and the number of cells transferred were higher for single-species (L96) than for multi-species biofilms (F96). These results are consistent with those reported by Pang and Yuk^23^, who found higher *L. monocytogenes* cell densities in single-species biofilms compared to dual-species biofilms formed with *P. fluorescens*. These authors attributed their findings to a competitive advantage of *P. fluorescens* over *L. monocytogenes*. Similarly, it could be that the co-existing microbiota competes with *L. monocytogenes* in F96, leading to a lower load than L96.

However, the TRs of F96 were higher than those of L96, suggesting the involvement of the coexisting microbiota in the potential for cross-contamination by *L. monocytogenes*. TR does not solely depend on the initial microbial load but also on the properties of the biofilm surface, which are highly conditioned by the content and composition of EPS, such as carbohydrates, extracellular DNA and lipids, which affect the viscoelasticity of biofilms^44^. As an example, a comparative study of EPS-producing and non-EPS-producing strains of *Staphylococcus epidermidis* showed that the number of cells transferred to nanopillared silicon surfaces was higher in the case of EPS-producing biofilms^45^. *L. monocytogenes* produces biofilms with a low EPS content, consisting mainly of proteins^46^. Unpublished results from our laboratory on single-species biofilms formed by various strains of *L. monocytogenes* confirm these findings. Thus, it would be expected that L96 would barely present EPS. On the contrary, bacteria that coexist in F96, such as *A. johnsonii* and *Rhodococcus* sp, are known to produce EPS and have a high biofilm-forming ability^47,48^. Although EPS were not analysed in the multi-species biofilms, the preponderance of EPS-producing strains would be expected to provide F96 with a viscoelasticity that would lead *L. monocytogenes* to detach from biofilms, enhancing the cross-contamination potential.

Few studies have conducted a comparative analysis of the contamination risk posed by *L. monocytogenes* from single-species versus multi-species biofilms, which limits direct comparisons with our present findings. One notable exception is the work of Pang and Yuk^23^, who investigated mixed-species biofilms of *L. monocytogenes* and *P. fluorescens*. The authors observed significantly higher TRs and numbers of transferred cells for single-species biofilms of *L. monocytogenes* than for mixed-species biofilms. However, their approach differed significantly from ours. We used a more complex biofilm system consisting of multiple bacterial species and employed experimental conditions—low inoculum and starvation—that mirrored the reality of FPEs.

Another issue of concern is the lack of knowledge regarding the behaviour of *L. monocytogenes* following cross-contamination of food from multi-species biofilms. *L. monocytogenes* can grow under refrigerated conditions; thus, challenge studies are essential to ensure the safety of RTE products. In the European Union, Regulation (EC) 2073/2005 mandates that RTE foods susceptible to *L. monocytogenes* growth must maintain a microbial load below 100 CFU/g throughout their shelf-life. In such products, the risk of listeriosis is highly dependent on the ability of *L. monocytogenes* to grow. Consequently, we conducted a study to investigate if there was an increased risk of *L. monocytogenes* growth during storage of cold-smoked salmon, whether it came from single- or multi-species biofilms.

It was thus found that *L. monocytogenes* cells from multi-species biofilms were more likely to thrive in vacuum-packaged cold-smoked salmon than those from single-species biofilms. It is considered that having previously been part of multi-species biofilms seems to confer a pre-adaptative advantage, enhancing *L. monocytogenes* growth. Whether this is also the case in other foods configured as different ecological niches is a question to be examined in the future. Consequently, when encountering the microbiota-rich environment of smoked salmon, it is better prepared to proliferate significantly. This environment is primarily dominated by lactic acid bacteria^49^, known producers of bacteriocins and other inhibitory metabolites^50,51^. *L. monocytogenes* cells from multi-species biofilms are likely better adapted to coexist with other bacteria, such as those in the salmon microbiota. Therefore, it is hypothesised that the co-existence of various bacterial species within multi-species biofilms may induce modifications in *L. monocytogenes*, conferring a competitive advantage that accelerates its growth relative to single-species biofilm-derived cells.

The growth differences observed between multi-species and single-species biofilm cells are particularly relevant within the context of European legislation. For this reason, we conducted a case study simulating a scenario where a single smoked salmon slice from an 80 g package was contaminated with *L. monocytogenes* from F96 or L96 biofilms. After 15 days of refrigerated storage, the *L. monocytogenes* load in samples contaminated from F96 exceeded the regulatory limit of 100 CFU/g. This underscores a significantly greater food safety risk and highlights the importance of considering multi-species biofilm contamination as real case scenarios that would then be incorporated into the design of more realistic and protective challenge studies, ultimately leading to more effective control of *L. monocytogenes*.

Furthermore, our results demonstrate the importance of using an experimental distribution-based stochastic approach, followed by robust statistical analysis, in studying the transfer dynamics of *L. monocytogenes* to food. It allows researchers to reproduce variable contamination scenarios that better reflect actual conditions in FPEs and thus capture key differences between biofilms that conventional methods may overlook.

This approach could also enable us to develop more accurate Quantitative Microbial Risk Assessments of *L. monocytogenes* in RTE food, particularly those that support its growth during their shelf-life (e.g. cold-smoked salmon). Challenge studies could thus move beyond deterministic inoculation schemes and instead model real-world cross-contamination risks based on observed stochastic dynamics. For food manufacturers, this would support more accurate microbial risk assessments and the development of tailored strategies to control *L. monocytogenes* that improve current food safety management systems. For example, manufacturers can prioritise sanitation efforts and monitoring in areas where multi-species biofilms are likely to form and enhance *L. monocytogenes* transfer. In addition, integrating these probabilistic contamination models into HACCP plans or predictive modelling tools can help set more protective critical limits and better manage residual risk in RTE products.

## Methods

### Bacterial strains and culture conditions

Bacterial strains under this study are listed in Table 2. The list comprises three *L. monocytogenes* strains, namely L1.96 (serogroup IIc, CC9), L1.107 (serogroup IIc, CC9) and L1.168 (serogroup IVb, CC217) and several representative strains of the most abundant genera coexisting with each of those strains of *L. monocytogenes*. All strains were isolated from three locations in FPEs where *L. monocytogenes* had been found, and were thus considered potential contamination sites^52^.

**Table 2:** Bacterial coexisting strains and *L. monocytogenes* are named according to the IIM-CSIC code

Biofilm	*L. monocytogenes*	Coexisting strains	Source
F96	L1.96	*Acinetobacter johnsonii* *Pseudoclavibacter helvolus* *Rhodococcus* sp. *Rothia* sp.	NFCS
F107	L1.107	*Acinetobacter johnsonii* *Pseudomonas putida* *Corynebacterium testudinoris*	FCS
F168	L1.168	*Chryseobacterium* sp. *Psychrobacter maritimus* *Psychrobacter cibarus*/sp.	NFCS

NFCS is the non-food contact surface; FCS is the food contact surface.

All strains were part of the Instituto de Investigaciones Marinas—Consejo Superior de Investigaciones Científicas (IIM-CSIC) culture collection, where stock cultures of each were maintained at −80 °C in sterile Brain-Heart Infusion Broth (BHI; Scharlab, Barcelona, Spain) with 50% (v v-1) glycerol. Short-term working cultures of each strain were stored at −20 °C under identical conditions. A maximum of 15 freeze-thaw cycles were permitted per cryovial. Strains were reactivated by adding 100 µL of working culture to 5 mL of Tryptone Soy Broth (TSB, Scharlab, Barcelona, Spain), followed by overnight incubation without shaking at 37 °C for *L. monocytogenes* strains, or 25 °C for the coexisting strains.

### Biofilm formation

All biofilms were formed under starvation conditions using TSB diluted at a 1:1000 ratio in Phosphate-Buffered Saline (PBS, Thermo Fisher Scientific, Massachusetts, USA). Multi-species biofilms F96, F107 and F168 were created by combining *L. monocytogenes* with the corresponding coexisting microbiota listed in Table 2. Single-species biofilms, L96, L107 and L168, were formed using each of the three *L. monocytogenes* strains separately.

The inoculum was individually standardised by adjusting reactivated cultures of each strain to an optical density (OD) at 700 nm of 0.100 ± 0.01, corresponding to ~10^8^ CFU/mL. Once adjusted, ten-fold serial dilutions were performed to obtain the desired microbial loads, which were verified in all cases by plating on Tryptone Soy Agar (TSA, Scharlab, Barcelona, Spain).

Biofilms were formed on sterile AISI 316 stainless steel coupons (1.0 mm thickness, 50 × 50 mm). Each coupon was placed into a plastic Petri dish (90 × 16.2 mm) with 25 mL of bacterial culture. Bacterial cultures contained ~10^2^ CFU/mL of *L. monocytogenes* and, in the case of multi-species biofilms, 10^5^ CFU/mL of each of the other bacteria in the consortium. Biofilms were cultured under static conditions for 72 h at 25 °C.

The concentration of *L. monocytogenes* in biofilms was determined using three biological replicates (coupons) in each trial. Once formed, biofilms were washed twice with 25 mL of PBS to remove non-attached cells. Subsequently, biofilms were sonicated for 2 min at 40 kHz and then scraped for 1 min to release the cells. Detached cells were serially diluted in peptone water (Scharlab, Barcelona, Spain), and appropriate serial dilutions were plated on *Listeria* Octavi Agostini Agar (ALOA, Scharlab, Barcelona, Spain) and cultured at 37 °C for 48 h.

### Transfer trials to smoked salmon fillets

Four independent transfer trials, unless otherwise stated, were conducted for each biofilm, using a minimum of 60 units of 80 g packs of sliced cold-smoked salmon in each trial. All packs were purchased from a local retailer and, where possible, were from the same production batch within each trial, with an expiry date of 9–15 days from purchase. Upon arrival at the laboratory, the packages were aseptically opened, and individual slices were trimmed into 4.5 cm diameter portions, each weighing ~5 g. In addition, slices were homogenised with peptone buffer at a 1:4 ratio in a stomacher blender, and aliquots of the homogenates were then plated onto ALOA agar to ensure that the load of *L. monocytogenes* was below the detection limit of 20 CFU/g.

**Fig. 9:**
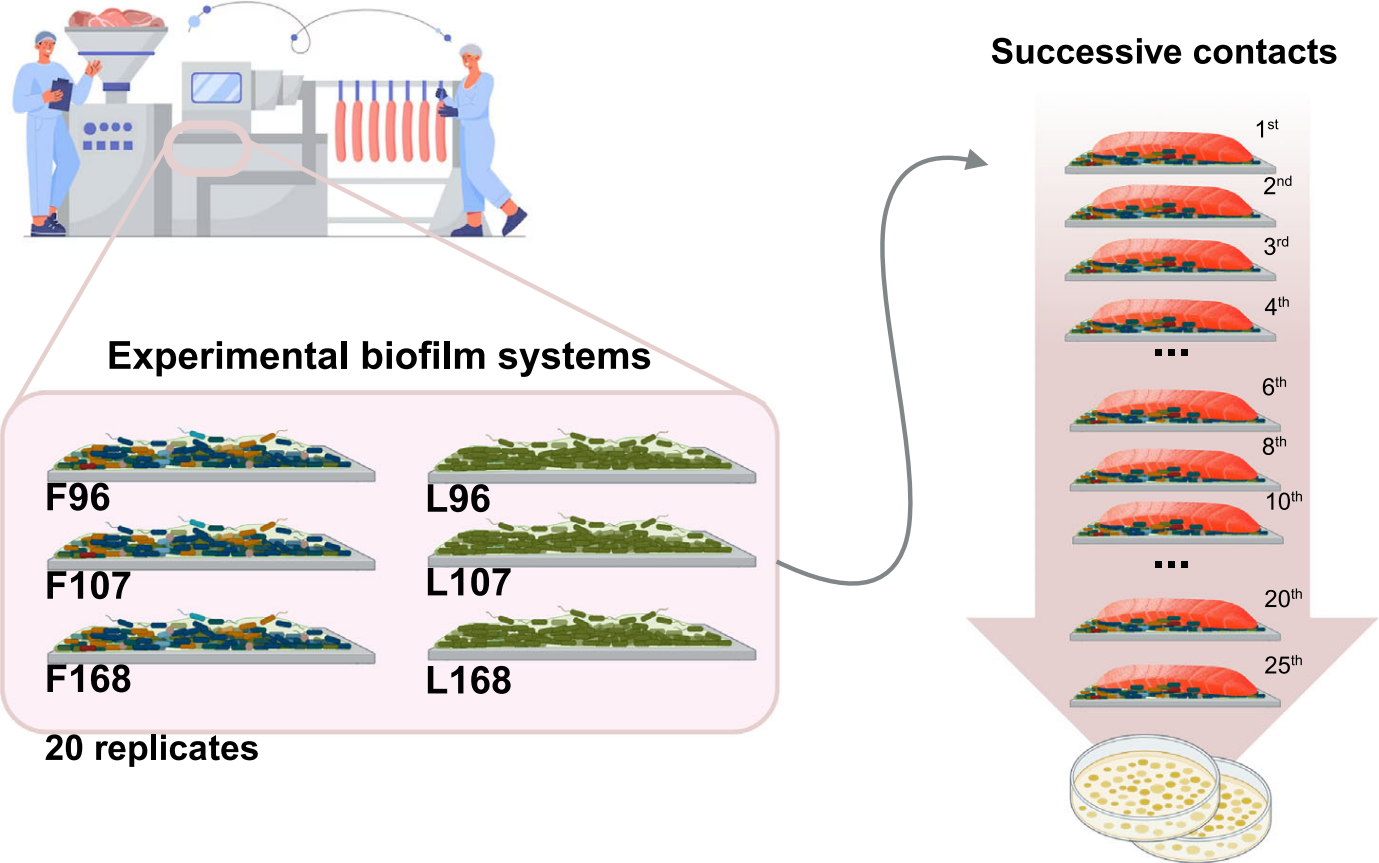
Experimental design for the transfer of *L.monocytogenes* from biofilms to cold-smoked salmon. Twenty replicates of single-species (L96, L107 and L168) and multi-species (F96, F107 and F168) biofilms were cultured and subsequently used for cell transfer to cold-smoked salmon. For each biofilm, 25 consecutive contacts were made to salmon fillets. *L.* *monocytogenes* loads on the salmon were quantified and reported as CFU/g. This image was created with BioRender.com.

Before transferring *L. monocytogenes* cells, the biofilms (coupons) were washed twice with PBS to remove non-attached cells. Subsequently, 25 portions of smoked salmon slices (5 g each) were sequentially contacted for 30 s with the biofilm surface, making 25 contacts per biofilm. Contacts were made by placing each portion on the biofilm without exerting pressure. Unless otherwise stated, four transfer trials were performed on different days for each biofilm, with five biological replicates (coupons) in each trial. Consequently, 20 distinct coupons were used to transfer biofilm cells to 25 portions of smoked salmon slices each, yielding 500 contact events per system. Figure 9 summarises the experimental process.

The number of *L. monocytogenes* cells transferred to the portions (CFU/g) corresponding to 1st, 2nd, 3rd, 4th, 6th, 8th, 10th, 15th, 20th and 25th contacts was determined by homogenising the portions in peptone buffer at a 1:4 ratio and plating homogenates in ALOA. Next, TRs were calculated for each of those contacts as follows:

1 $${TR}\,\left( \% \right)=100* \left(\frac{{Number\; of\; transferred\; cells}}{{Number\; of\; biofilm\; cells}}\right)$$

The number of cells of *L. monocytogenes* transferred to 5 g of smoked salmon is expressed as CFU/g, whereas the number of biofilm cells of *L. monocytogenes* is expressed as total CFU per coupon (Supplementary Table 1). The latter was determined by multiplying the number of *L. monocytogenes* cells per cm^2^ of coupon surface area (CFU/cm^2^) by the surface area of the coupon in contact with the salmon slice (i.e. 15.90 cm^2^), as not all the coupon surface area (25 cm^2^) was in contact with the slice.

### Storage in vacuum package trials

*L. monocytogenes* L1.96 cells were transferred from 72 h biofilms, both single (L96) and multi-species (F96), to smoked salmon slices as previously described. Portions contaminated during the first and sixth contacts were vacuum-packed in plastic bags and stored at 5 °C for 15 days. The *L. monocytogenes* load in the salmon slices (CFU/g) was reported at the initial time point (day 0) and after 7 and 15 days of storage by plating on ALOA. This experiment was performed in triplicate.

### Data analysis and modelling

Data analysis was conducted using R software version 4.3.0^53^ and RStudio version 4.1.3^54^. All graphs were generated using *ggplot2*^55^. The Shapiro-Wilk test was used to check data normality, and Levene’s test was used to determine the homogeneity of variances. Significant differences were determined via one-way ANOVA, followed by Tukey’s HSD test post hoc in normally distributed data. The Kruskal–Wallis rank sum test was used non-normally, followed by Dunn’s post hoc test.

Experimental TR data for each biofilm were first fitted to the exponential, logistic and Weibull distributions using the *fitdistrplus* package^56^. This initial step aimed to identify how well each standard distribution could describe the variability observed in raw, ungrouped TR data from successive transfer events. Then, a Monte Carlo method was applied to generate 10,000 synthetic row datasets to improve the estimates of the best fit distribution (Weibull) and mitigate the impact of possible outliers and unstable data points. Each synthetic row (y_i_) was based on experimental data (x_i_) by adding white noise sampled from a normal distribution with a mean of zero and a standard deviation proportional to the original data. Column-wise (contact) means were calculated, obtaining a new representative row vector (y), a robust approximation of the original data distribution. However, the added noise was substantial due to large standard deviations. A Weibull distribution was fitted to (y) to reduce noise, resulting in a new vector (w). The error (e) between $w$ and y was calculated by the root of the sum of squared differences and then subtracted from each row vector of the expanded database, yielding an adjusted data matrix, where each row is denoted as ${z}_{i}=\left|{y}_{i}-e\right|$. Subsequently, column-wise means were recalculated, and a new Weibull distribution was fitted to the resulting adjusted row vector ($z$). Once the bias correction was applied, it was confirmed to be sufficient through empirical simulations, as established in statistical references^57^.

Next, TR values from different contacts that showed no significant statistical differences (*p* > 0.05, Table 2) were grouped into clusters via Hierarchical Clustering, under the rationale that these sets could represent a more homogeneous contamination dynamic. For these clustered datasets, we shifted the fitting to gamma, log-normal, and Weibull distributions (fitdistrplus package). The KS test was used to assess the statistical differences between the frequency distributions obtained for each cluster. The optimal parameters of each fitted distribution were then used to calculate probabilities via the empirical cumulative distribution function from the stats package^53^.

## Supplementary information

Supplementary materials

## Data Availability

The authors declare that all data supporting the findings of this study are available in the paper, and the statistical analysis code will be made available upon reasonable request to the corresponding author.
