# Supplementary material for: A formal analysis of Listeria monocytogenes cross-contamination dynamics in multi-species biofilms
Source: NPJ Sci Food. 2025 Aug 8;9:168. doi: 10.1038/s41538-025-00535-3 (PMC12332031; doi:10.1038/s41538-025-00535-3)
Supplement: Supplementary file 1 — Supplementary materials [file 41538_2025_535_MOESM1_ESM.pdf]

Supplementary Table 1. L. monocytogenes *cell counts in the initial biofilm (CFU/cm^2 and total CFU), and recovered after transfer to the smoked-salmon slices (CFU/g) on the successive contacts (1st – 25th). Total CFU was calculated by multiplying the initial CFU/cm^2 by the surface of contact of the salmon slice (15.904 cm^2). No cell counts were recorded for single-species biofilms L107 and L168.*

	**L96**			**F96**			**F107**			**F168**		
	**Mean ± SD**	**Min**	**Max**	**Mean ± SD**	**Min**	**Max**	**Mean ± SD**	**Min**	**Max**	**Mean ± SD**	**Min**	**Max**
CFU/cm^2	5.65E+05 ± 3.36E+05	2.28E+05	1.07E+06	4.79E+04 ± 3.65E+04	1.04E+04	9.13E+04	1.18E+05 ± 8.76E+04	3.60E+03	2.29E+05	4.79E+05 ± 2.09E+05	1.80E+05	8.70E+05
Total CFU	5.20E+06 ± 2.30E+06	3.31E+06	9.15E+06	4.15E+05 ± 2.39E+05	1.65E+05	7.85E+05	1.87E+06 ±1.39E+06	5.72E+04	3.64E+06	7.61E+06 ± 3.32E+06	2.86E+06	1.38E+07
**CFU/g**												
1st	1.02E+06 ± 8.04E+05	1.30E+05	2.41E+06	5.37E+04 ± 4.36E+04	1.25E+04	1.45E+05	2.09E+05 ± 1.81E+05	4.74E+03	6.28E+05	4.97E+05 ± 2.85E+05	1.71E+05	1.10E+06
2nd	1.08E+06 ± 9.84E+05	1.50E+05	2.97E+06	3.13E+04 ± 4.25E+04	3.19E+03	1.84E+05	1.57E+05 ± 1.41E+05	2.16E+03	4.72E+05	3.31E+05 ± 1.93E+05	6.05E+04	8.30E+05
3rd	8.77E+05 ± 1.01E+06	7.79E+04	3.02E+06	1.74E+04 ± 1.68E+04	1.15E+03	5.13E+04	8.71E+04 ± 7.43E+04	1.85E+03	2.64E+05	1.91E+05 ± 9.61E+04	2.85E+04	4.12E+05
4th	3.29E+05 ± 2.84E+05	4.77E+04	9.90E+05	9.73E+03 ± 1.02E+04	6.51E+02	3.34E+04	3.64E+04 ± 3.03E+04	2.40E+02	1.13E+05	9.65E+04 ± 6.02E+04	2.75E+04	2.45E+05
6th	1.57E+05 ± 1.62E+05	1.67E+04	5.32E+05	4.27E+03 ± 4.48E+03	5.78E+01	1.39E+04	2.54E+04 ± 2.37E+04	2.04E+02	8.96E+04	4.97E+04 ± 1.88E+04	1.38E+04	8.33E+04
8th	9.24E+04 ± 8.55E+04	1.50E+04	2.59E+05	3.26E+03 ± 3.68E+03	5.39E+01	1.14E+04	1.37E+04 ± 1.17E+04	4.16E+02	4.13E+04	4.06E+04 ± 5.75E+04	3.77E+03	2.79E+05
10th	7.10E+04 ± 6.70E+04	3.36E+03	1.90E+05	2.83E+03 ± 4.96E+03	1.92E+01	2.12E+04	9.18E+03 ± 7.63E+03	2.13E+02	2.45E+04	2.55E+04 ± 3.28E+04	3.73E+03	1.59E+05
15th	2.10E+04 ± 2.08E+04	1.87E+03	6.36E+04	1.05E+03 ± 1.46E+03	2.45E+01	6.38E+03	3.70E+03 ± 3.50E+03	2.09E+01	1.27E+04	7.23E+03 ± 9.35E+03	1.80E+03	4.28E+04
20th	6.68E+03 ± 6.78E+03	7.80E+02	2.17E+04	3.70E+02 ± 3.61E+02	0.00E+00	1.37E+03	1.81E+03 ± 1.69E+03	1.96E+01	5.76E+03	4.22E+03 ± 5.73E+03	1.37E+02	2.67E+04
25th	6.06E+03 ± 8.00E+03	8.64E+01	2.41E+04	1.82E+02 ± 2.78E+02	0.00E+00	9.87E+02	1.45E+03 ± 1.85E+03	0.00E+00	6.27E+03	1.69E+03 ± 1.69E+03	4.57E+02	8.08E+03

Supplementary Table 2. *Goodness-of-fit (GOF) analysis for exponential, logistic and Weibull models to the experimental transfer rates of single-species (L96) and multi-species (F96, F107, and F168).*

Biofilm	Stats	Exponential	Logistic	Weibull
**L96**	**GOF statistic**			
	Kolmogorov-Smirnov test	0.26	0.24	0.17
	Anderson-Darling test	1.64	0.99	0.34
	**GOF criteria**			
	Akaike information criterion	41.37	55.04	40.17
**F96**	**GOF statistic**			
	Kolmogorov-Smirnov test	0.31	0.26	0.15
	Anderson-Darling test	1.39	0.98	0.17
	**GOF criteria**			
	Akaike information criterion	50.56	65.96	49.19
**F107**	**GOF statistic**			
	Kolmogorov-Smirnov test	0.27	0.26	0.11
	Anderson-Darling test	1.42	1.00	0.19
	**GOF criteria**			
	Akaike information criterion	45.78	61.09	44.59
**F168**	**GOF statistic**			
	Kolmogorov-Smirnov test	0.27	0.25	0.12
	Anderson-Darling test	1.43	0.91	0.18
	**GOF criteria**			
	Akaike information criterion	51.41	66.16	50.01

Supplementary Table 3. *Weibull model parameters of shape and scale, mean ± SD, for single-species (L96) and multi-species (F96, F107 and F168) biofilms. There are no significant differences between biofilms for shape or scale values.*

Biofilm	Shape	Scale
L96	0.61 ± 0.06	1.92 ± 1.22
F96	0.58 ± 0.15	2.68 ± 1.70
F107	0.63 ± 0.10	2.23 ± 0.67
F168	0.62 ± 0.09	2.93 ± 1.23

Supplementary Table 4. *Goodness-of-fit (GOF) analysis for log-normal, gamma and Weibull frequency distributions to the experimental transfer rates of single-species (L96) and multi-species (F96, F107 and F168) biofilms.*

Biofilm	Stats	Log-normal	Gamma	Weibull
**L96**	**Cluster 1**			
	**GOF statistic**			
	Kolmogorov-Smirnov test	0.09	0.12	0.13
	Anderson-Darling test	0.56	0.89	1.03
	**GOF criteria**			
	Akaike information criterion	302.76	302.91	304.21
	**Cluster 2**			
	**GOF statistic**			
	Kolmogorov-Smirnov test	0.10	0.05	0.06
	Anderson-Darling test	0.47	0.23	0.23
	**GOF criteria**			
	Akaike information criterion	140.98	138.30	138.56
	**Cluster 3**			
	**GOF statistic**			
	Kolmogorov-Smirnov test	0.07	0.12	0.09
	Anderson-Darling test	0.33	0.86	0.48
	**GOF criteria**			
	Akaike information criterion	132.82	139.73	133.24
**F96**	**Cluster 1**			
	**GOF statistic**			
	Kolmogorov-Smirnov test	0.07	0.05	0.07
	Anderson-Darling test	0.37	0.36	0.41
	**GOF criteria**			
	Akaike information criterion	525.71	523.24	525.82
	**Cluster 2**			
	**GOF statistic**			
	Kolmogorov-Smirnov test	0.13	0.08	0.09
	Anderson-Darling test	2.111	0.51	0.61
	**GOF criteria**			
	Akaike information criterion	257.14	240.01	240.61
	**Cluster 3**			
	**GOF statistic**			
	Kolmogorov-Smirnov test	0.15	0.07	0.08
	Anderson-Darling test	0.80	0.94	3.41
	**GOF criteria**			
	Akaike information criterion	121.43	144.15	146.11

Biofilm	Stats	Log-normal	Gamma	Weibull
**F107**	**Cluster 1**			
	**GOF statistic**			
	Kolmogorov-Smirnov test	0.07	0.09	0.09
	Anderson-Darling test	0.27	0.65	0.97
	**GOF criteria**			
	Akaike information criterion	412.55	417.83	423.73
	**Cluster 2**			
	**GOF statistic**	**Log-normal**	**Gamma**	**Weibull**
	Kolmogorov-Smirnov test	0.08	0.07	0.07
	Anderson-Darling test	0.49	0.59	0.68
	**GOF criteria**			
	Akaike information criterion	205.03	206.84	210.26
	**Cluster 3**			
	**GOF statistic**			
	Kolmogorov-Smirnov test	0.11	0.15	0.12
	Anderson-Darling test	0.17	0.42	0.18
	**GOF criteria**			
	Akaike information criterion	190.50	211.83	212.96
**F168**	**Cluster 1**			
	**GOF statistic**			
	Kolmogorov-Smirnov test	0.06	0.10	0.10
	Anderson-Darling test	0.29	0.57	0.86
	**GOF criteria**			
	Akaike information criterion	401.54	401.62	404.68
	**Cluster 2**			
	**GOF statistic**	**Log-normal**	**Gamma**	**Weibull**
	Kolmogorov-Smirnov test	0.05	0.09	0.11
	Anderson-Darling test	0.31	1.03	1.31
	**GOF criteria**			
	Akaike information criterion	249.81	255.91	259.15
	**Cluster 3**			
	**GOF statistic**			
	Kolmogorov-Smirnov test	0.07	0.12	0.11
	Anderson-Darling test	0.34	1.63	1.64
	**GOF criteria**			
	Akaike information criterion	101.18	116.05	120.01

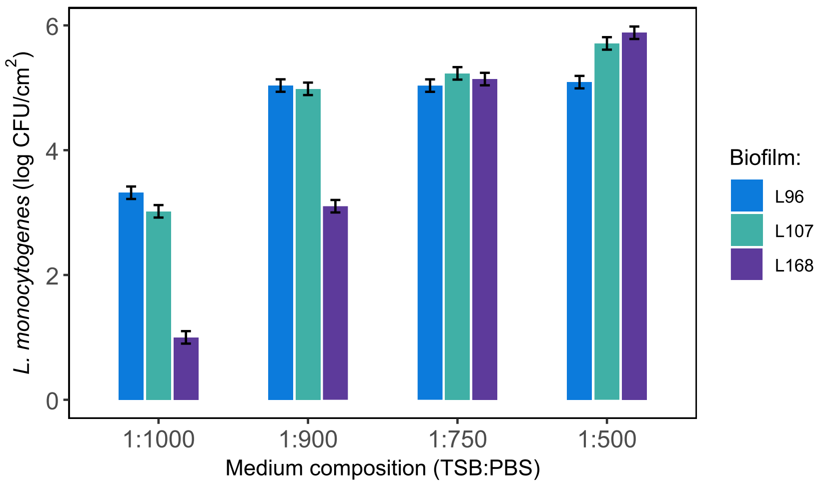

***Supplementary Figure 1**. L. monocytogenes cell counts for single-species biofilms L96, L107 and L168 in low nutrient media TSB:PBS.*
